# Concepts of Regeneration for Spinal Diseases in 2022

**DOI:** 10.3390/ijms23179710

**Published:** 2022-08-26

**Authors:** Takashi Yurube, Inbo Han, Daisuke Sakai

**Affiliations:** 1 Department of Orthopaedic Surgery, Graduate School of Medicine, Kobe University, 7-5-1 Kusunoki-cho, Chuo-ku, Kobe 650-0017, Japan; 2 Department of Neurosurgery, School of Medicine, CHA Bundang Medical Center, CHA University, Seongnam-si 13496, Korea; 3 Department of Orthopedic Surgery, School of Medicine, Tokai University, 143 Shimokasuya, Isehara 259-1193, Japan

## 1. Introduction

It is our pleasure to announce the publication of the Special Issue “Regeneration for Spinal Diseases 2.0” in the *International Journal of Molecular Sciences* (ISSN 1422-0067). Spinal diseases place a significant burden on the general population. The morbidity of degenerative spinal diseases, including intervertebral disc degeneration and osteoporosis, is quite high and increases with age, and the affected population may suffer from a long-term disability. Furthermore, the most serious spine-related conditions can reach spinal cord injury due to traumatic or non-traumatic causes. Despite recent advances in the management of these prevalent but problematic spinal pathologies, there is a growing research interest in discovering novel therapeutic strategies.

Our previous *International Journal of Molecular Sciences* Special Issue “Regeneration for Spinal Diseases” in 2021 was highly successful, as 15 articles with rigorous scientific backgrounds were published. Because of the success of the first edition, we would like to add further results and new perspectives from more recent research projects to the second edition. This *International Journal of Molecular Sciences* Special Issue “Regeneration for Spinal Diseases 2.0” in 2022 includes 11 cutting-edge original research articles consisting of 6 papers concerning degenerative disc disease, 3 concerning spinal cord injury, 1 concerning adolescent idiopathic scoliosis, and 1 concerning spinal fusion surgery. Moreover, three expertized review articles summarizing regenerative treatment approaches for the intervertebral disc were compiled. All of these 14 articles should provide helpful insights for the current clinical management and future basic science development of regenerative treatment strategies for intractable spinal diseases.

These articles were published in the *International Journal of Molecular Sciences* (https://www.mdpi.com/journal/ijms, 15 August 2022), Section: Molecular Pathology, Diagnostics, and Therapeutics (https://www.mdpi.com/journal/ijms/sections/Pathology_Diagnostics_Therapeutics, 15 August 2022), Topic: Regeneration for Spinal Diseases 2.0 (https://www.mdpi.com/journal/ijms/special_issues/Spinal_Diseases_2, 15 August 2022). Articles from the previous edition of this Special Issue were also published in the *International Journal of Molecular Sciences*, Section: Molecular Pathology, Diagnostics, and Therapeutics, Topic: Regeneration for Spinal Diseases (https://www.mdpi.com/journal/ijms/special_issues/Spinal_Diseases, 15 August 2022). The *International Journal of Molecular Sciences* is an international, peer-reviewed, open-access journal published by the Multidisciplinary Digital Publishing Institute, universally and freely accessible online.

## 2. Degenerative Disc Disease

With the global trend of aging, low back pain is a worldwide health problem because of the enormous morbidity and socioeconomic strain. The cause of low back pain is largely non-specific; however, lumbar disc degeneration is one of the independent risk factors. Intervertebral disc degeneration increases with age, which can develop not only back pain but also neurological disorders, including radiculopathy, myelopathy, and paralysis. The current primary treatment for degenerative disc disease is surgical resection and/or fusion, resulting in the loss of load function, shock absorption, and spinal movement. Therefore, the development of regenerative therapies for disc degeneration is highly demanded.

The intervertebral disc has a complex structure, consisting of the central nucleus pulposus (NP), peripheral annulus fibrosus (AF), and sandwiching cartilage endplates. The disc is the largest avascular, hypoxic, low-nutrient organ in the body. Particularly in the NP compared to the AF, cells depend on the diffusion from blood vessels at the disc margins to obtain oxygen and nutrients. Blood supply reduction, subchondral bone sclerosis, and endplate calcification, all occurring as a result of mechanical stress, injury, smoking, radiation, and aging, can thus limit the transport to the disc. This additional loss of oxygen and nutrient supply is a suspected initiator of intervertebral disc degeneration.

Kim JW et al. [1] developed a nondegradable form under normoxia of hypoxia-inducible factor-1alpha (HIF-1α) by point mutation, transfected it to human disc NP cells, and presented apoptosis suppression and upregulated glycolysis-involved and matrix synthesis-related gene expression. In vivo intradiscal transfection of this constitutively active HIF-1α reduced needle puncture-induced radiological and histological degeneration with maintained collagen type II expression. Future therapeutic strategies can be developed based on the importance of HIF-1α to protect against disc degeneration.

Croft AS et al. [2] examined effects of different mechanical loading regimes on ex vivo bovine caudal disc organ cultures. These applications showed the loss of disc height, cell viability, and glycosaminoglycan content, corresponding with the intensity of complex dynamic compression and torsion. Moreover, mechanosensitive *cartilage intermediate layer protein* gene was downregulated overall, while Hippo signaling pathway-involved *mammalian STE20-like protein kinase 1* gene was upregulated in the high stress regime. Excessive loading can accelerate disc degeneration, possibly through Hippo signaling.

Zhong J et al. [3] reported responses of human and rat disc AF cells to ionizing radiation on inducing cellular senescence. Radiation exposures reached substantial senescence induction with increased cyclin-dependent kinase inhibitors p16 and p21 expression, catabolic enzyme expression, and anabolic aggrecan degradation products. Whole-body ionizing radiation-exposed mice demonstrated similar findings of cellular senescence and matrix catabolism in vivo. These results shed light on the pathomechanism of disc degeneration, as well as potential adverse effects of ionizing radiation on spinal health.

Miyagi M et al. [4] studied the relationship between clinical and radiographic degeneration and pain-related molecule expression in human symptomatic degenerative lumbar discs surgically collected. Axial loading and mechanical stress due to high body mass index and positive vacuum phenomenon correlated with the upregulation of *calcitonin gene-related peptide* (*CGRP*) and *microsomal prostaglandin E synthase-1* (*mPGES-1*). While *mPGES-1* expression correlated with *CGRP* and *NGF* expression, *NGF* expression correlated with *tumor necrosis factor-alpha* and *interleukin* (*IL*)*-6* expression. Pain-related *CGRP* and *mPGES-1* are possibly associated with chronic discogenic low back pain.

Kusakabe T et al. [5] studied the intracellular signaling network between NGF, prostaglandins (PGs), and mitogen-activated protein kinases (MAPKs) in human degenerative discs. Disc cell NGF induction by IL-1beta (IL-1β) was inhibited by PGE_2_ or PGE_1_. The PGE_2_ and PGE_1_ supplementation additionally increased IL-1β-induced expression of dual-specificity phosphatase (DUSP)-1, a negative regulator of MAPK signaling, but decreased IL-1β-induced MAPK phosphorylation. However, in DUSP-1 knockdown cells, IL-1β-induced MAPK phosphorylation and NGF expression were further enhanced. Thus, PGE_2_ and PGE_1_ inhibit IL-1β-induced NGF expression through DUSP-1-mediated MAPK suppression, suggesting DUSP-1 as a new therapeutic target molecule for low back pain.

Seki S et al. [6] explored the differentiation of normal adult human dermal fibroblasts into disc NP-like cells by the ectopic expression of *MYC proto-oncogene*, *KLF transcription factor 4*, *notochord homeobox*, *SRY-box transcription factor* (*SOX*) *5*, *SOX6*, and *SOX9*. The applied three-dimensional alginate bead culture system and transforming growth factor-beta (TGF-β) signaling could be required for the in vitro direct differentiation and reprograming. Consequently, the induced NP-like cells presented a fully differentiated phenotype, suggesting as a cell source target for the treatment of intervertebral disc disease.

In the papers reviewing current strategies for intervertebral disc regeneration, Herger N et al. [7] hypothesized that pain relief and disability-related improvements by intradiscal mesenchymal stem cell (MSC) injection would result from the immunomodulatory potential rather than the regenerative properties. Hence, the application of intradiscal MSC injection to degenerative discs of patients with Modic type 1 pathologic inflammatory, fibrotic changes in the vertebral bone marrow was extensively reviewed. Consequently, MSCs might represent a safe and multimodal treatment approach with promising immunomodulatory and regenerative properties. Intradiscal MSC-based therapy could prevent the inflammatory disc–bone marrow crosstalk and repair Modic type 1 changes. To facilitate this, the condition of cartilage endplates is essential.

Bhujel B et al. [8] explored whether pleiotropic effects of MSCs would be related to the differentiation capacity or mediated by the secretion of soluble paracrine factors, MSC-derived exosomes. Their extensive literature search and review clarified diverse prospects of MSC-derived exosomes to repair degenerative discs. The MSC-derived exosomes could promote cell proliferation, inhibit apoptotic cell death, reduce inflammation, and maintain extracellular matrix homeostasis in the intervertebral disc. However, the safety of MSC-derived exosomes still remains uncovered.

Kasamkattil J et al. [9] focused on spheroids for cell-based disc therapies, which are three-dimensional multicellular aggregates with the architecture supporting cell differentiation, extracellular matrix synthesis, cell–matrix interaction, adhesion enhancement, and microenvironment improvement. Spheroids could be applied to the disc, both in scaffold-free and scaffold-based configurations, possibly providing advantages over cell suspensions. In disc NP restoration, spheroids combined with injectable biomaterials are recommended to generate the volume. In disc AF restoration, spheroids could serve as building blocks for living patches, together with sealing biomaterials. This review highlights the potential of spheroid-based tissue engineering strategies for disc repair and regeneration.

## 3. Spinal Cord Injury

Spinal cord injury is a devastating condition of the damage to the spinal cord, primarily caused by trauma but also by vascular disease, infection, and tumors, which can induce temporary or permanent motor, sensory, and autonomic dysfunction and associated complications. Currently, effective treatments are limited, which are largely symptom relieving and deterioration preventing, e.g., corticosteroid administration (controversial), decompression and/or stabilization, and rehabilitation. Therefore, there is a great need to develop new therapeutic strategies for neurological recovery and neural repair.

Song BG et al. [10] hypothesized that synaptic cell adhesion molecules (SynCAMs) would facilitate the adhesion between axons and astrocytes and glial scar formation. Thus, a loss-of-function study of SynCAMs was designed using SynCAM3-knockout mice subjected to spinal cord injury. Reduced glial scar formation and transformation of reactive astrocytes into scar-forming astrocytes, as well as improved locomotor functional recovery and extracellular matrix reconstitution, were observed in SynCAM3-knockout mice. Consequently, SynCAM3 could be a novel therapeutic target for spinal cord injury.

Kim Y et al. [11] tested bazedoxifene acetate (BZA), a third-generation selective estrogen receptor modulator, for neuroprotection and remyelination using neural, macrophage, and endothelial cells and a rat spinal cord injury model. In vitro, anti-inflammatory and blood–spinal cord barrier integrity-preserving effects of BZA were observed. In vivo, anti-apoptosis, anti-inflammation with reduced MAPK phosphorylation and IL-6 expression, enhanced remyelination, and improved locomotor functional recovery by BZA were detected. Thus, BZA could be a potential therapeutic agent for spinal cord injury.

Kim CK et al. [12] studied effects of adult human neural stem cells (ahNSCs), derived from the temporal cortex of focal cortical dysplasia type IIIa surgical specimens, on spinal cord injury in rats. Following ahNSC transplantation into the lateral ventricle of rats, improved motor functional recovery as well as increased spread, regenerated nerve fibers with a closer distance between neuronal nuclei in damaged spinal cord tissues were observed. These neuroprotective effects of ahNSCs were mediated by anti-apoptosis of spinal cord neurons and paracrine factors driven by monocyte chemoattractant protein-1, suggesting as a future effective and safe cell therapy for spinal cord injury.

## 4. Adolescent Idiopathic Scoliosis

Adolescent idiopathic scoliosis is a three-dimensional spinal curvature that progresses during the pubertal growth spurt. The prevalence of scoliosis is approximately 0.5–4%, being much higher in girls. The progression of the scoliotic curvature and associated deformity can lead to back pain and respiratory dysfunction. If the curvature progresses > 25° in skeletally immature patients, bracing is warranted. Surgery is recommended at >45° curvature to halt the progression of scoliosis and related problems. The development of scoliosis treatment based on the pathomechanism is necessary.

Seki S et al. [13] investigated the molecular pathomechanism of scoliosis progression, focusing on the hypertrophy of the ligamentum flavum. In this study, the comparison between experimental and control specimens from the same donor is valuable to exclude the individual difference. The ligamentum flavum hypertrophy with the increased collagen fiber density was more remarkable on the convex side than on the concave side. Biologically, *ELKS/RAB6-interacting/CAST family member 2* and *MAF bZIP transcription factor B* genes were associated with the ligamentum flavum hypertrophy through increased *TGF-β1* and *IL-6* expression in patients with adolescent idiopathic scoliosis. These findings are important to consider the progression of scoliosis.

## 5. Spinal Fusion Surgery

As a result of developmental, degenerative, and traumatic spinal disorders, unstable spondylosis, spondylolisthesis, spondylolysis, spinal deformity, and spinal fractures can cause severe nociceptive back pain and intractable neuropathic complications. Spinal fusion surgery is a surgical intervention to stabilize segments, including discs and facet joints, as well as to decompress neural tissues, including the dural tube and nerve roots, ultimately providing acceptable clinical and radiological outcomes. To achieve spinal fusion, the use of autologous bone graft is the gold standard. However, due to the limitation of the supply and morbidity associated with autograft harvest, various bone graft materials, including allografts, demineralized bone matrix, and synthetic bone substitutes, including ceramics and cements, combined with growth factors, have been developed.

Kwon SY et al. [14] examined the contribution of whitlockite, the second most abundant inorganic component (approximately 25%) of human bones, to bone remodeling and formation in a mouse spinal fusion model. In vitro, the whitlockite-implanted group presented more homogeneous, smaller grains with nanopores (<500 nm) than other groups of conventional ceramics, including hydroxyapatite and beta-tricalcium phosphate. In vivo, the whitlockite-implanted group showed larger fusion mass formation and better graft incorporation into the decorticated mouse spine with higher immunopositivity for osteocalcin, osteopontin, and CD31. These findings support the potential of whitlockite as an alternative bone substitute with an improved bone conductivity.

## 6. Conclusions

In conclusion, we would like to declare future regathering and recomposing further successful, updated Special Issues, e.g., “Regeneration for Spinal Diseases 3.0”.
